# Accumulation of CCR4^+^ CTLA-4^hi^ FOXP3^+^CD25^hi^ Regulatory T Cells in Colon Adenocarcinomas Correlate to Reduced Activation of Conventional T Cells

**DOI:** 10.1371/journal.pone.0030695

**Published:** 2012-02-01

**Authors:** Helena Svensson, Veronica Olofsson, Samuel Lundin, Chakradhar Yakkala, Stellan Björck, Lars Börjesson, Bengt Gustavsson, Marianne Quiding-Järbrink

**Affiliations:** 1 Department of Microbiology and Immunology, Institute of Biomedicine, University of Gothenburg, Göteborg, Sweden; 2 Department of Surgery, Institute of Clinical Sciences, University of Gothenburg, Göteborg, Sweden; City of Hope National Medical Center and Beckman Research Institute, United States of America

## Abstract

**Background:**

Colorectal cancer usually gives rise to a specific anti-tumor immune response, but for unknown reasons the resulting immunity is not able to clear the tumor. Recruitment of activated effector lymphocytes to the tumor is important for efficient anti-tumor responses, while the presence of regulatory T cells (Treg) down-modulate tumor-specific immunity. We therefore aimed to determine homing mechanisms and activation stage of Treg and effector T cell infiltrating colon tumors compared to cells from the unaffected mucosa in patients suffering from colon adenocarcinoma.

**Methodology/Principal Findings:**

Lymphocytes were isolated from unaffected and tumor mucosa from patients with colon adenocarcinoma, and flow cytometry, immunohistochemistry, and quantitative PCR was used to investigate the homing mechanisms and activation stage of infiltrating Treg and conventional lymphocytes. We detected significantly higher frequencies of CD25^high^FOXP3^+^CD127^low^ putative Treg in tumors than unaffected mucosa, which had a complete demethylation in the FOXP3 promotor. Tumor-associated Treg had a high expression of CTLA-4, and some appeared to be antigen experienced effector/memory cells based on their expression of αEβ7 (CD103). There were also significantly fewer activated T cells and more CTLA-4^+^ conventional T cells susceptible to immune regulation in the tumor-associated mucosa. In contrast, CD8^+^granzyme B^+^ putative cytotoxic cells were efficiently recruited to the tumors. The frequencies of cells expressing α4β7 and the Th1 associated chemokine receptor CXCR3 were significantly decreased among CD4^+^ T cells in the tumor, while frequencies of CD4^+^CCR4^+^ lymphocytes were significantly increased.

**Conclusions/Significance:**

This study shows that CCR4^+^CTLA4^hi^ Treg accumulate in colon tumors, while the frequencies of activated conventional Th1 type T cells are decreased. The altered lymphocyte composition in colon tumors will probably diminish the ability of the immune system to effectively attack tumor cells, and reducing the Treg activity is an important challenge for future immunotherapy protocols.

## Introduction

Colorectal cancer (CRC) is one of the most common malignant diseases with an annual incidence of 945 000 cases worldwide and an annual mortality of about 500 000 [1]. Cytotoxic T lymphocytes (CTL), supported by cytokine-producing Th1 cells, are one of the most important effectors mechanisms in immunity against tumors [2]. However, to be effective, tumor-specific lymphocytes must be able to leave the circulation and enter into the tumor. Lymphocyte extravasation from blood to tissues is a multi-step process involving transient interactions between selectins and their carbohydrate antigens, followed by chemokine binding to specific receptors and integrin activation, leading to firm arrest and transendothelial migration [3], [4]. Integrin α_4_β_7_ is the major mediator of lymphocyte homing to healthy gastrointestinal tissues and binds to mucosal addressin cellular adhesion molecule-1 (MAdCAM-1), expressed on endothelial cells in the gastrointestinal tract [5]. L-selectin binds to peripheral node addressin (PNAd), expressed by high endothelial venules in secondary lymphoid tissues, and mediates homing of naïve and central memory cells to these tissues [6], [7]. In addition, chemokine production in the tissue also contributes to differential tissue distribution of migrating lymphocytes.

Tumors have developed several mechanisms by which they can escape the host immune response [8]. The induction of so-called regulatory T cells (Treg) in many types of cancer disease probably also contribute to tumor immune evasion [9], [10], [11]. Treg are suppressive CD4^+^CD25^hi^FOXP3^+^CD127^low^ T cells, acting on conventional T cells in a cell-cell contact-dependent manner [12]. The precise mechanisms of Treg mediated suppression have not yet been fully characterized, but the suggested mediators include CTLA-4 and Granzyme B [13], [14]. Furthermore, the tumor microenvironment may influence endothelial differentiation and function [15], [16] and we have recently shown a shift in endothelial adhesion molecule expression in gastric adenocarcinomas. The tumor-associated mucosa had a decrease in MAdCAM-1^+^ and increase in PNAd^+^ blood vessels compared to in the unaffected mucosa and also increased production of the CCR4 ligand CCL17. These findings were correlated to increased frequencies of L-selectin^+^CCR4^+^ Treg and fewer activated lymphocytes in the tumor-associated mucosa [17], [18].

Treg have also been shown to accumulate in colon tumors, especially those associated with microsatellite instability, and in the circulation of patients with CRC [19], [20], [21], [22], [23], [24], and several studies actually show that high intratumoral Treg frequencies or low CD3/FOXP3 ratios correlate with improved prognosis [19], [25], [26], [27], [28]. This is in contrast to most other tumor types, where increased Treg frequencies correlate with a poor prognosis [9], [10], [11]. To elucidate the effect of Treg accumulation on local immune cell function and recruitment, we explored the activation stage, as well as the homing mechanisms of lymphocytes in human colon adenocarcinomas, and compared that to the adjacent unaffected mucosa. Our results show that CCR4^+^CTLA4^hi^ Treg accumulate in colon tumors, while the frequencies of activated conventional Th1 type T cells are decreased. The altered lymphocyte recruitment to colon tumors will probably contribute to the ability of the tumor to avoid immune mediated elimination.

## Results

### Lymphocyte subsets in tumors and unaffected colonic mucosa

Lamina propria lymphocytes (LPL) were isolated from tumor and unaffected mucosa from patients undergoing colectomy, and analysis of surface molecules defining T cells, B cells, and NK cells was carried out using flow cytometry. The distribution of CD4^+^ and CD8^+^ T cells was similar in the two types of tissue, both total frequencies and frequencies of CD45RO^+^ memory subsets. Similar and low frequencies of CD56^+^CD3^−^ NK cells were also detected. In contrast, CD19^+^ B cells were significantly decreased in the tumor compared to the unaffected colonic mucosa (p<0.01, data not shown).

**Figure 1:**
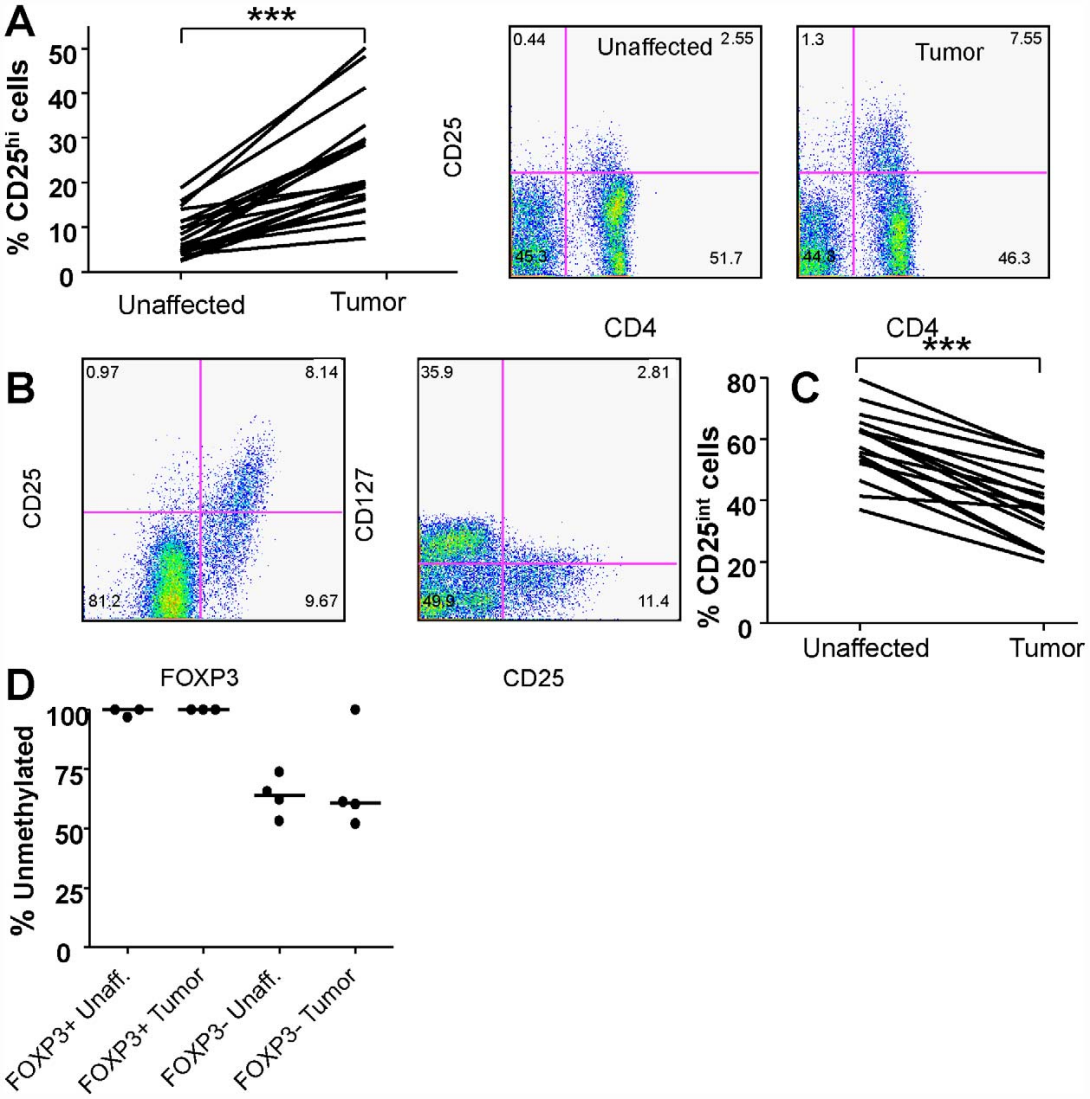
Treg frequencies in tumor and unaffected mucosa. The frequencies of CD4^+^CD25^high^ putative Treg in tumor associated and unaffected mucosa from colon cancer patients were investigated using flow cytometry. (A) Paired individual frequencies of CD4^+^CD25^hi^putative Tregs among all CD4^+^ T cells from 17 patients, and an example of CD4 and CD25 staining in one individual. (B) Intracellular FOXP3 and surface CD127 staining of CD25^hi^ cells in the tumor from one individual. (C) Frequencies of CD4^+^CD25^int^ T cells among all CD4^+^ T cells in unaffected and tumor mucosa. Connected lines represents data from the same individual. (D) Methylation status of the FOXP3 promoter region in FOXP3^+^ and FOXP3^−^ CD4^+^ T cells sorted by flow cytometry from lamina propria cells isolated from unaffected and colon tumor tissue, as determined by Ms-SNUPE. Each symbol represents data from one individual. *** p<0.001, Wilcoxon signed rank test.

To examine the presence of putative Treg we analyzed the frequencies of CD25^high^ cells in the CD4^+^ LPL populations. The frequencies of CD4^+^CD25^high^ T cells in the tumors were significantly higher (p<0.001, fig. 1A) compared to unaffected colonic mucosa from the same patients. The CD25^high^cells were invariably FOXP3^+^ and CD127^low^, which to date is the best combination of markers to identify putative Treg [29] (Fig. 1B). Since FOXP3 can be transiently expressed by human conventional T cells following stimulation, we analyzed if the colonic FOXP3^+^ cells were stably expressing FOXP3, by assessing the methylation status of the FOXP3 promoter region using methylation-sensitive single nucleotide primer-extension analysis (Ms-SNUPE). There was an almost complete demethylation of the FOXP3 promoter in the CD4^+^FOXP3^+^ cells from both the tumor and unaffected tissue (Fig. 1D), indicating that they stably express FOXP3. CD4^+^FOXP3^−^ cells from both unaffected colonic tissue and tumors had a lower demethylation than the CD4^+^FOXP3^+^ cells (range 51–100% and 53–74% in the tumor and unaffected tissue, respectively; Fig. 1D).

### Localization of T cells in tumor and unaffected mucosa

**Figure 2:**
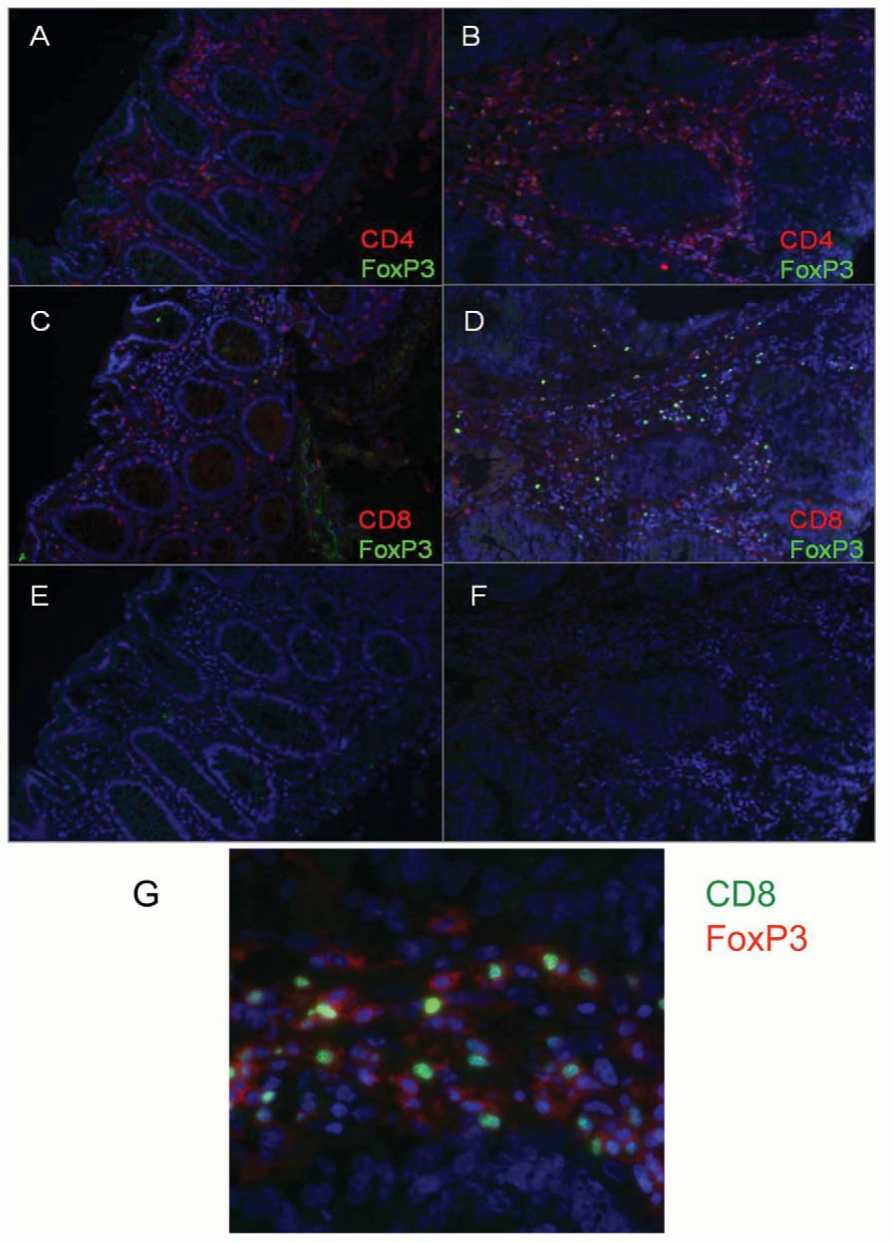
Immunofluorescence detection of T cell subsets in tumor and unaffected mucosa. The expression of surface CD4 (A and B) and CD8 (C and D) in combination with intracellular FOXP3 was determined in unaffected (A and C) and tumor mucosa (B and D). E and F show isotype control staining of the respective tissues, and G a close-up of FOXP3^+^ cells (green nucleus) in close association with CD8^+^ conventional T cells (red surface staining) in a section of tumor mucosa.

We also visualized CD4^+^ and CD8^+^ T cells as well as Treg in tumor and unaffected mucosa *in situ* using immunofluorescence. The unaffected mucosa had large numbers of CD4^+^ cells scattered throughout the lamina propria (Fig. 2A), whereas the submucosa was clear of immune cells. CD8^+^ cells were present in lower numbers and seen in both the lamina propria and in-between epithelial cells (Fig. 2C). Treg were present in low numbers in the lamina propria. In the tumor, T cells were seen in lamina propria-like parts of the tissue, where CD4^+^, CD8^+^ and Tregs were co-localized (Fig. 2B and D). On the other hand, all three T cell populations only rarely infiltrated among the tumor cells. Treg were more frequent in the tumor tissue than unaffected tissue, and sometimes closely associated with CD4^+^ or CD8^+^ cells, possibly executing their regulatory effect (Fig. 2G).

### Activation and differentiation state of LPL from tumor and unaffected mucosa

**Figure 3:**
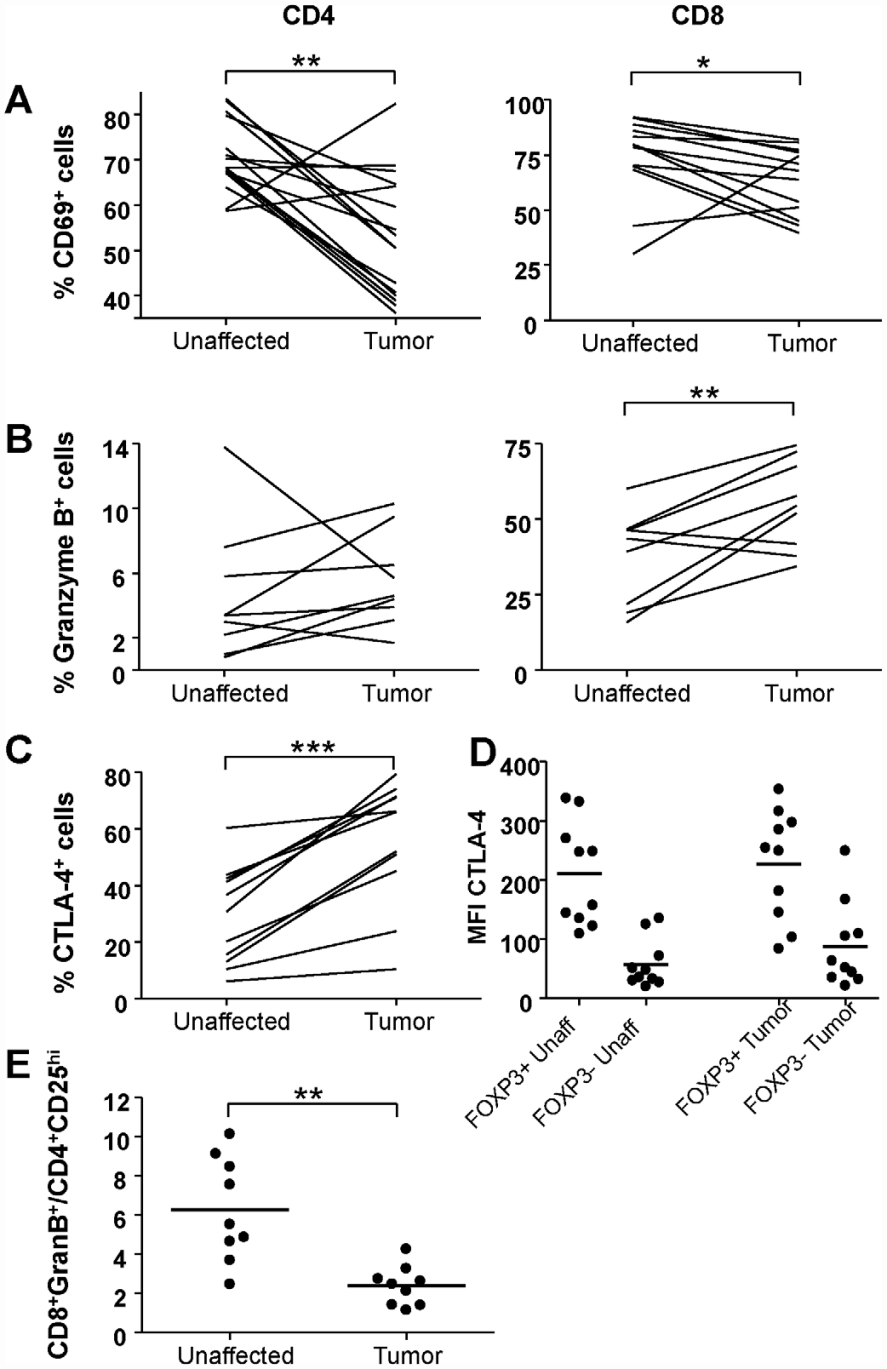
Activation and differentiation state of T cells from tumor and unaffected mucosa. Left hand panels in A–B show CD4^+^ T cells and right hand panels show CD8^+^ T cells. The expression of surface CD69 (A) and intracellular Granzyme B (B) was evaluated on conventional CD4^+^ FOXP3^−^ and CD8^+^ T cells from the tumor and unaffected mucosa using flow cytometry. The frequencies of positive cells are given as the percentage of all CD4^+^ or CD8^+^ cells, respectively. The expression of intracellular CTLA-4 (C) was evaluated in conventional CD4^+^ FOXP3^−^ T cells and the frequencies of positive cells are given as the percentage of all CD4^+^ T cells. C. (D) MFI for CTLA4 on conventional CD4^+^FOXP3^−^ T cells and putative Tregs isolated from tumors and unaffected tissue. Graphs show paired individual values from 9 to 15 patients. (E) Ratios of CD8^+^Granzyme B^+^ cells to CD4^+^CD25^hi^ Treg cells among cells isolated from tumor and unaffected mucosa * p<0.05, ** p<0.01, *** p<0.001, Wilcoxon signed rank test.

To determine activation and differentiation stage of the T cells present in tumors and unaffected tissue, flow cytometry was used to analyse expression of CD45RA, CD69, CTLA-4, and Granzyme B on freshly isolated LPL. Recently activated CD69^+^ T cells were relatively frequent in the unaffected lamina propria, but present in significantly lower numbers in the tumor (Fig. 3A). There were also higher frequencies of CD4^+^CD25^int^ activated cells in the unaffected than in the tumor-associated mucosa (Fig. 1A and C). The distribution of naïve, CD45RA^+^, CD4^+^ and CD8^+^ cells was similar in the tumor and the surrounding mucosa (data not shown). However, higher frequencies of CD8^+^Granzyme B^+^ cells, presumably differentiated CTL, were present in the tumor than the unaffected tissue (Fig. 3B). Still, the ratio of CD8^+^Granzyme B^+^ cells to CD4^+^CD25^hi^ Treg cells was significantly higher (p<0.01) in the unaffected then the tumor mucosa (Fig. 3E). Granzyme B has also been described as an effector molecule used by Treg [14], but in our material conventional CD4^+^ lymphocytes expressed low levels of Granzyme B (Fig. 3B), and Treg were completely devoid of Granzyme B expression.

CTLA-4 has been identified as an effector molecule mediating suppression by Tregs [13], and virtually all Treg in the tumors did express CTLA-4, while conventional CD4^+^ T cells had a lower and more varied expression of CTLA-4 (Fig. 3C). The mean fluorescence intensity (MFI) showed that Treg had more CTLA-4 per cell than conventional CD4^+^ T cells, both in the tumor and in the unaffected tissue (Fig. 3D). On conventional T cells, CTLA-4 is increased in the later stages of T cell activation, and mediates down-regulated activity of the T cell expressing it [30]. There was, however, no significant difference in the CTLA-4 expression by tumor derived conventional CD4^+^ T cells than by corresponding cells from unaffected tissue (Fig. 3D).

### Lymphocyte and endothelial adhesion molecules in tumor and unaffected mucosa

**Figure 4:**
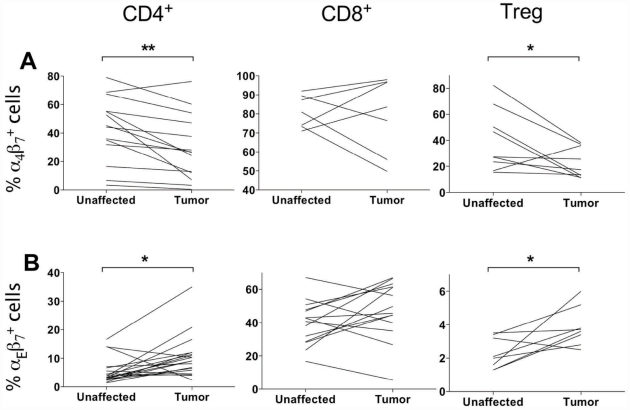
Expression of β7 integrins on T cells from tumor and unaffected mucosa. The expression of α4β7 (A) and αEβ7 (B) was evaluated on CD4^+^ and CD8^+^ T cells and Treg from the tumor and unaffected mucosa using flow cytometry. The frequencies of positive cells are given as the percentage of all CD4^+^ or CD8^+^ cells, respectively. Graphs show paired individual values from 7 to 16 patients. * p<0.05, ** p<0.01, Wilcoxon signed rank test.

To better understand the homing mechanisms involved in T cell migration to colon tumors we examined homing receptor expression on CD4^+^, CD8^+^ LPL and Treg. These studies showed a significantly decreased (p<0.05) expression of the mucosal homing receptor α4β7 on CD4^+^ cells in tumor tissue compared to unaffected tissue (Fig. 4A). On the other hand, integrin αEβ7, that serves to attach lymphocytes to E-cadherin on epithelial cells, but also has been implicated in Treg function [31], [32], was expressed by more CD4^+^ tumor infiltrating T cells than CD4^+^ T cells in unaffected mucosa (p<0.05). The CD8^+^ T cell expression of αEβ7 was much higher than in CD4^+^ T cells, probably reflecting the eventual intraepithelial position of many CD8^+^ T cells. Interestingly, there was also a significant (p<0.05) increase in αEβ7 expression on tumor infiltrating Treg compared to Treg in unaffected tissue (Fig. 4B), even though Treg were never seen in the epithelium by IHC.

L-selectin is used as a homing receptor for secondary lymphoid organs, but is also upregulated on T cells migrating to gastric tumors [17]. In colon cancer patients, however, there was no consistent difference in the frequencies of L-selectin^+^ conventional T cells or Treg in the tumor and in the surrounding tissue (data not shown).

**Figure 5:**
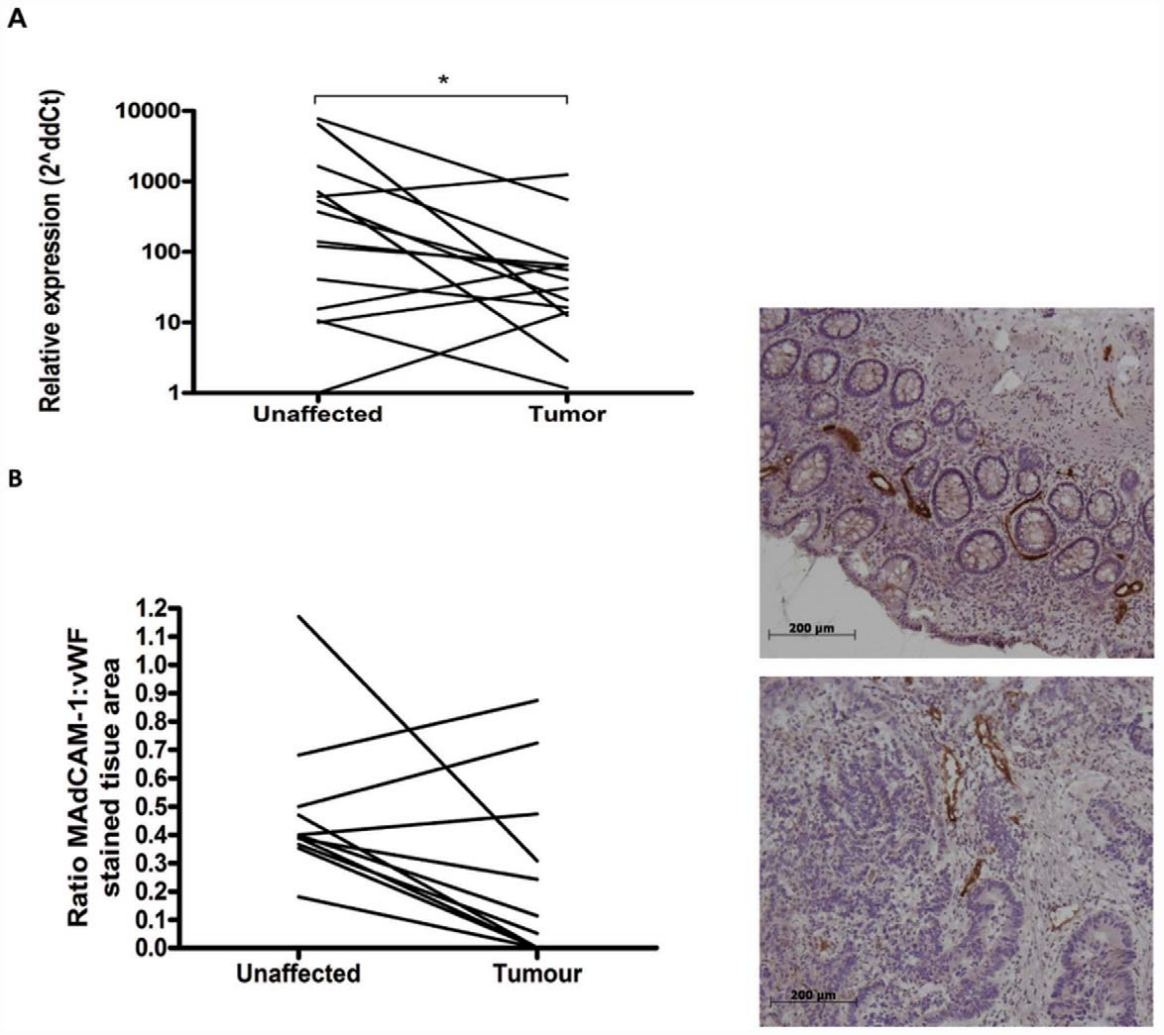
Expression of MAdCAM-1 on endothelial cells in tumor and unaffected mucosa. The expression of MAdCAM-1 mRNA (A) and protein (B) was evaluated in the tumor and unaffected mucosa real time PCR and immunohistochemistry respectively. The mRNA concentrations were normalized against 18s mRNA. Graphs show paired individual values from 8 and 12 patients, and representative MAdCAM-1 staining in unaffected mucosa (above) and tumor tissue (below). * p<0.05, Wilcoxon signed rank test.

Real time PCR demonstrated that MAdCAM-1 expression was significantly reduced in the tumor compared to the unaffected tissue (Fig. 5A). These results were confirmed by immunohistochemistry, showing a lower density of MAdCAM-1^+^ vessels in the tumor-associated mucosa (Fig. 5B). PNAd, on the other hand could not be detected at all in the colon lamina propria, neither in tumor nor in the unaffected mucosa.

### Chemokine receptor expression on LPL from tumor and unaffected mucosa

**Figure 6:**
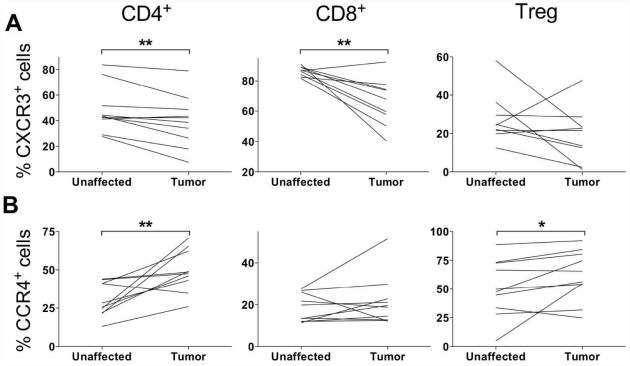
Chemokine receptor expression on T cells from tumor and unaffected mucosa. The expression of CXCR3 (A) and CCR4 (B) was evaluated on conventional CD4^+^ and CD8^+^ T cells and Treg from the tumor and unaffected mucosa using flow cytometry. The frequencies of positive cells are given as the percentage of all CD4^+^ or CD8^+^ cells, respectively. Graphs show paired individual values from 9 to 10 patients. * p<0.05, ** p<0.01, Wilcoxon signed rank test.

Flow cytometry analyses showed that CXCR3, mainly expressed by Th1 cells and CTL, was present on significantly fewer CD4^+^ and CD8^+^ LPL (p<0.01) in the tumor tissue compared to the unaffected tissue (Fig. 6), whereas in the case of Treg there was only a trend towards decreasing frequencies of CXCR3^+^ cells. In contrast, CCR4, a chemokine receptor associated with Th2 and Treg responses, was reciprocally expressed, i.e. there was a higher frequency of CCR4^+^ cells in CD4^+^ conventional T cells and Treg in the tumor than in the unaffected mucosa (Fig. 6). Finally, the chemokine receptor CCR5, which is usually co-expressed with CXCR3 on Th1 cells [33] was expressed in similar frequencies in tumor and unaffected mucosa. This was also true for CXCR4, CCR7, CCR9 and CCR10 (data not shown).

### Chemokine concentrations in unaffected and tumor mucosa

Finally, we examined the presence of chemokines signaling through CCR4 and CXCR3 in protein extracts from tumor and unaffected tissue to investigate if differences in chemokine production could explain the altered T cell recruitment to tumor tissues. Among the CCR4 binding chemokines, CCL17 (TARC) concentrations were not significantly different while the concentration of CCL22 (MDC) was significantly higher in tumors than in unaffected tissues (304±320 pg CCL22/mg total protein and 124±111 pg/mg in tumor and unaffected tissue, respectively, p<0.01), a finding that could explain the increased number of CCR4^+^ T cells in the tumor tissue. Among the CXCR3 binding chemokines, CXCL9 (MIG) concentrations did not differ between unaffected and tumor tissue. Surprisingly, CXCL10 (IP-10) and CXCL11 (I-TAC) were both present in significantly higher concentrations in the tumor tissue compare to unaffected mucosa (70±56 and 8.2±6.6 pg CXCL10/mg protein, respectively, p<0.01 and 672±442 and 211±161 pg CXCL11/mg protein, respectively, p<0.001) even though both CXCR3^+^ CD4^+^ and CD8^+^ T cells were present in significantly lower numbers in the tumor compared to the unaffected tissue.

## Discussion

Immunosuppressive Treg have been described in colon cancer patients, and their numbers are increased in the tumor compared to the surrounding unaffected mucosa [20], [21], [22], [23]. However, several recent studies show that high Treg frequencies actually correlate with a better prognosis in colon cancer patients [19], [25], [26]. We therefore aimed to define the subsets and putative functions of Treg and conventional T cells that had migrated to the tumor-associated colonic mucosa as well as the homing mechanisms used by these cells. There was no change in the frequencies of the CD4^+^ or CD8^+^ T lymphocytes and NK cells between tumor-associated and unaffected mucosa, while the frequencies of CD19^+^ B cells were lower in the tumor. However, T cell immunity is generally held to be more important for anti-tumor responses than B cells [2], and we therefore analyzed the activation stage of tumor-infiltrating T cells. Thus, we could demonstrate reduced levels of recently activated CD69^+^ T cells and CD25^int^CD4^+^ T cells from tumor-associated colonic mucosa compared to unaffected mucosa. A population of spleenic CD4^+^CD69^+^, but CD25^−^ and FoxP3^−^, T cells with regulatory activity has been described in tumor-bearing mice [34]. If a corresponding population exists in human tumors, it would be concealed among the CD4^+^FOXP3^−^CD69^+^ T cells, but as this population is substantially decreased in the tumors compared to the unaffected mucosa, it may not be the most prominent local Treg population. The reduced frequencies of CD69^+^ and CD25^int^ T cells were was accompanied by a significant increase in CD4^+^CD25^high^FOXP3^+^CD127^low^ Treg, confirming several recent studies that used immunohistochemistry or PCR techniques to detect Treg [19], [21], [22], [23]. We could, however, extend the previous findings by a more detailed characterization of tumor associated Tregs. Thus, we demonstrate a complete demethylation of the FOXP3 promoter in colon Treg from tumor tissue, suggesting that the FOXP3^+^ cells stably express FOXP3 as part of an established Treg phenotype, and not due to recent activation of conventional T cells.

Furthermore, some of the tumor-associated Treg express αEβ7. This integrin is used to anchor intraepithelial T cells to E-cadherin on epithelial cells [31], but has also been implicated in Treg function. A recent study by Siewert *et al* [32] shows that αEβ7^+^ Tregs are antigen experienced effector/memory cells with a high turn-over rate. Such cells can develop in the periphery, and we therefore speculate that the αEβ7^+^ Treg we detected may arise locally in the tumor. Our results are in contrast to results from a murine colon cancer model where approximately 90% of the tumor-associated Treg expressed αEβ7 [35]. However, frequencies may vary due to species differences or the fact that the colon tumor cells were injected subcutaneously in the murine model. Finally, there was a high expression of CTLA-4 on Treg in the human colon tumors. CTLA-4 on Treg is crucial for suppression of autoimmunity and for efficient anti-tumor responses [13]. Therefore, the presence of CTLA-4 on tumor Treg would indicate active suppression of the anti-tumor immune response. Our observations that Treg can be found in close proximity to FOXP3^−^CD4^+^ and CD8^+^ T cells in the tumor tissue and the lower frequencies of activated conventional T cells in tumors would also support this conclusion. It is also worth noting that increased Treg frequencies were found in all tumors, irrespectively of their differentiation grade and stage, as well as localization. Taken together with the general view that there are several variants of colon adenocarcinoma with different characteristics accumulating in different parts of the colon [36], this indicates that increased Treg frequencies is truly a general finding in all types of colon cancer.

Recent studies, including more than 2 000 patients, have shown that there is actually a positive correlation between higher Treg frequencies in the tumor and increased survival [19], [26], [27]. However, when patients were grouped according to microsatellite instability caused by deficiencies in mismatch repair (MMR) genes, the positive correlation of Treg infiltration to survival in multivariate analyses only remained in MMR-proficient patients [26]. Furthermore, it was recently shown that high Treg frequencies in colon tumors before treatment correlate to a better overall survival and a better response to chemo- or chemoimmunotherapy [25]. These findings are not easily reconciled with our observations indicating that Treg in colon tumors are active immunosuppressive cells, and also with several other studies showing that Treg infiltration in other types of solid tumors correlate with poor prognosis [9], [10], [11]. However, in other studies, Treg frequencies did not correlate to patient outcome in colon cancer [28], [37]. Instead, the ratio of CD3^+^ lymphocytes to FOXP3^+^ Treg was a better predictor of disease-free survival than tumor stage or lymph node spread [28], and high intratumoral CD8^+^ to Treg ratios also correlate to better survival [37]. Thus, the ratio of Treg∶effector T cells may be a better measure of the net outcome of the immune response than just Treg frequencies alone, as also suggested in gastric tumors [38]. The exact tissue localization of Treg may also be important, since the location of tumor infiltrating lymphocytes relative to the invading margin and center of the tumor has been shown to be of prognostic value [39]. The patient material in the current study was relatively small, and did not reveal any correlation between Treg frequencies and survival during a 2–4.5 year follow-up period (data not shown).

In murine systems, a fraction of tumor-associated Tregs express Granzyme B, and this molecule also appears to contribute to Treg mediated suppression [14]. In the present study, however, there was no expression of Granzyme B at all in the Treg. On the other hand, the presence of putative cytotoxic Granzyme B^+^CD8^+^ T cells was increased in the tumor, and these cells may be a sign of ongoing anti-tumor responses. Still, the ratio of CD8^+^Granzyme B^+^ cells to CD4^+^CD25^hi^ Treg cells was higher in the unaffected then the tumor mucosa. In parallel to the increase in putative effector CD8^+^ CTL, there was an increase in CTLA-4^+^ cells within the CD4^+^ conventional T cells. These cells are susceptible to inhibition of effector functions through ligation of their CTLA-4. Thus, several suppressive and anti-tumor responses seem to exist side by side in the tumor, and the balance between them may decide the final outcome of the tumor disease.

Even though the homing mechanisms used by lymphocytes to enter healthy intestinal mucosa are relatively well-known, little is known about lymphocyte homing to tumors in the gastrointestinal tract. We could demonstrate that there are differences in T lymphocyte homing mechanisms between tumor-associated mucosa and unaffected mucosa in the colon. Previously, we have shown that gastric cancer patients have a decreased expression of MAdCAM-1 on tumor blood vessels, and that this was accompanied by fewer α_4_β_7_^+^ LPL in tumor mucosa compared to unaffected mucosa [17]. Our present results extend these findings to another major type of gastrointestinal tumors, showing that also in colon tumors there are decreased frequencies of CD4^+^ conventional T cells and Treg expressing α_4_β_7_ and a concomitant decrease in MAdCAM-1 expression on the blood vessels. In contrast to gastric cancer [18], we could never detect the L-selectin ligand PNAd in the colon tissue.

Not only selectins and integrins contribute to lymphocyte homing to mucosal surfaces, but the expression of chemokine receptors on the lymphocyte surface is crucial for lymphocyte homing. The chemokine receptor CXCR3 is expressed on activated Th1-type lymphocytes and CTL and is abundant on lymphocytes in the mucosa of normal intestine [40]. We show here that the frequencies of CXCR3^+^ conventional T cells are significantly reduced in tumor compared to unaffected mucosa from the same patient. CD4^+^ Th1 lymphocytes expressing CXCR3 have been shown to be important for anti-tumor responses [41], [42], and the reduction in CXCR3^+^ cells probably mirrors a reduction of Th1 cells and CD8^+^ CTL in the tumor area [43], [44]. The tumor evasion from CXCR3^+^ cells is probably not dependent on chemokine production by the tumor since CXCR3 ligands CXCL10 and CXCL11 both are present in higher concentrations in the tumor. The reduction in CXCR3^+^ cells in the tumor stand out in comparison with expression of many other chemokine receptors, which were similar in the tumor and unaffected mucosa. However, Treg and conventional CD4^+^ T cells expressing CCR4, which is primarily expressed on Th2 type CD4^+^ T cells and Treg [18], [33], [45] were accumulating in the colon tumors. Our previous study also showed increased CCR4 expression on gastric tumor-associated Treg, accompanied by increased expression of CCR4 ligands [18]. Here, significantly increased tissue concentrations of the CCR4-ligand CCL22 were also found in colonic tumors, and probably contribute to recruitment of CCR4^+^ cells. Together, these data suggest a selective exclusion of CXCR3^+^ conventional T cells from the tumor-associated mucosa, with a concomitant recruitment of CCR4^+^ Treg and conventional CD4^+^ T cells.

In conclusion, this study show accumulation of CCR4^+^CTLA-4^hi^ Treg stably expressing FOXP3 in colon adenocarcinomas, and close contact between Treg and conventional CD4^+^ and CD8^+^ T cells. In parallel, frequencies of activated CD4^+^ conventional T cells with a Th1 profile were decreased in the tumor, while granzyme B^+^ putative cytotoxic CD8^+^ cells were present. The altered recruitment of Treg and conventional T cells was probably dependent on changes in endothelial MAdCAM-1 expression and tissue production of CCL22. Taken together, this change of the local immunological balance favors suppressive mechanisms and will probably diminish the ability of the immune response to effectively attack the tumor.

## Materials and Methods

### Volunteers and collection of specimens

Thirthy-one patients undergoing partial colectomy at Sahlgrenska University Hospital due to colon adenocarcinomas were included in the study. Patient data is presented in table 1. The study was approved by the Regional Board of Ethics in Medical Research in west Sweden, and all volunteers gave a written informed consent before participation. None of the patients had undergone radiotheraphy or chemotheraphy for at least three years prior to colectomy. During or immediately after colectomy, a strip of tumor tissue was collected together with unaffected mucosa, collected at least five centimeters away from the tumor. Small pieces of the tissue were collected in RNAlater (Applied Biosystems, Foster City, CA, USA) for isolation of RNA, embedded in OCT for subsequent immunohistochemical analysis, or snap frozen for protein extraction. Remaining tissue samples were transported in cold PBS for isolation of LPL within one hour. Since varying numbers of LPL could be retrieved from the tissue from different patients, all analyses could not be performed in all patients. In no case were patients eliminated from the assays, solely the availability of cells determined which assays were performed.

**Table 1:** Characteristics of the colon cancer patients included in the study.

		females	males
	n	14	17
	age	41–95	57–86
Tumor location	ascending	8	12
	transverse	3	1
	descending	3	4
Differentiation grade	high	-	1
	medium	8	12
	low	3	1
	mucinous	2	1
Tumor stage	T1	-	2
	T2	1	4
	T3	10	5
	T4	3	5
Lymph node spread		6	4
Distant metastases		1	1

Information on tumor stage, differentiation grade and metastases was retrieved from the routine pathology report. Patients were followed for 22 to 54 months after surgery (mean follow-up time 32 months) and recurrence of the cancer disease or cancer related death was recorded.

### Isolation of LPL

LPL were isolated using collagenase/DNase enzymatic digestion after removal of epithelial cells, as previously described [46]. This method gives an optimal yield of LPL with only small amounts of epithelium remaining and preserves the surface markers analysed in the study. The cell yield varied from person to person, and therefore, all analyses could not be performed in all individuals.

### Flow cytometry analysis of intracellular and cell surface markers

LPL from tumor and tumor-free mucosa were incubated with fluorescently labeled antibodies for 30 min at 4°C. Fluorescent antibodies used were labelled with PerCP/PECy5 (CD4, CD19), APC (CD8, CD69), FITC (CD103, CD45RA, CD8, CXCR3, CD127) and PE (L-selectin, CD25, CCR4, CD56, CCR5), and were all from BD Biosciences (Erembodegem, Belgium). Integrin α4β7 was detected with biotinylated mAb ACT-1 (kindly provided by Dr. D. Picarella, Millenium Inc, Cambridge, MA). For intracellular staining, surface markers were first labelled, the cells fixed and then intracellular staining of FOXP3 and CTLA-4 was performed using permeabilisation buffer (eBioscience, Hatfield, UK) according to the protocol provided by the manufacturer. After staining, cells were fixed in BD cellFIX^TM^ (BD Biosciences) and analysis was carried out on a FACS Calibur using FlowJo 8.1.1 software.

### RNA isolation and quantitative real time RT-PCR

Biopsies were homogenized by grinding with a pellet pestle (Sigma-Aldrich) in an eppendorf tube in the presence of lysis buffer (RNeasy Mini Kit, Qiagen, Solna, Sweden). The homogenized tissue was added to a QIAshredder column (Qiagen) and total RNA was extracted according to manufactures descriptions. Extracted RNA was run on an 1% agarose gel to check RNA integrity and concentration was measured on a NanoDrop ND-1000 spectrophotometer, software version 2.5.1 and stored at −80°C until further use.

Preparation of cDNA was performed using the Omniscript kit (Qiagen, Germany). For each 20 µl reaction, 500 ng total RNA was used. Gene expression assays for MAdCAM-1 and 18s mRNA were obtained from Applied Biosystems. For calculating the difference in expression we used the comparative CT (threshold cycle) method, also called 2^−ΔΔCt^. Data are presented as fold change in mRNA expression, normalized to the 18s mRNA

### Immunohistochemical detection of endothelial adhesion molecules

For analysis of endothelial MAdCAM-1, PNAd and von Willebrand factor (vWF) expression, 8 µm tissue sections were cut onto glass slides and fixed in ice-cold acetone. Sections were rehydrated in cold PBS followed by blocking with an avidin/biotin kit (Molecular Probes, Eugene, OR, USA), and endogenous peroxidase by glucose oxidase (Sigma-Aldrich). Sections were incubated in PBS with anti-MAdCAM-1 (clone 10G3, kindly donated by Professor Sirpa Jalkanen, University of Turku), anti-pNAD (clone MECA-79, BD Biosciences), anti-vWF (clone F8/86, DAKO, Glostrup, Denmark) or isotype control for 1 hour at room temperature. Sections were then incubated with rabbit anti mouse Ig-HRP (DAKO) or with biotinylated mouse anti rat IgM (BD Biosciences) followed by avidin-HRP (ABC Elite, Vector, Burlingame, CA, USA). All sections were developed with DAB (Vector), counterstained in Meyer's hematoxyline and mounted in Mountex. Images were acquired using Zeiss Axiovision 4.7.2 and analyzed in Biopix iQ 2.1.5.

### Immunofluorescence detection of T cells in tissue sections

T cells were detected in tissue sections by double staining with anti-FOXP3 and anti-CD4 or CD8. Briefly, frozen sections were fixed in 4% paraformaldehyde, washed with PBS containing 0.1% saponin and endogenous biotin blocked as above. Thereafter, slides were incubated with mouse anti-FOXP3 (clone 236A/E7) [47] or isotype control (DAKO). Signal was detected and amplified using tyramide-Alexa fluor 488 amplification (TSA kit, Molecular probes). Sections were then incubated with mouse anti-CD4 or CD8 (DAKO) followed by detection with goat-anti-mouse IgG1 Alexa Fluor 594 (Molecular Probes) and mounted using gold-anti-fade mounting medium containing DAPI (Molecular Probes).

### *FOXP3* methylation analysis

LPL isolated from tumor and unaffected mucosa from 4 male patients were stained for flow cytometric analysis of CD4 and FOXP3 and sorted using a FACSAria cell sorter (BD) into CD4^+^FOXP3^+^ and CD4^+^FOXP3^−^ populations. Genomic DNA from these cells was isolated and bisulphite converted using an EZ DNA methylation kit (ZYMO research, Orange, CA). The FOXP3 promoter region was amplified by PCR, and the PCR product was then subjected to methylation-sensitive single nucleotide primer-extension analysis (Ms-SNUPE) by using an ABI prism SNaP shotTM multiplex kit (Applied Biosystems) as recently described [48]. Finally, the samples were subjected to capillary electrophoresis on an ABI prism 3730 genetic analyzer, and the results analyzed by using the Gene mapper V3.7 software.

### Protein extraction and detection of chemokines

Proteins were extracted from biopsies as previously described [49]. CXCL11, CCL17, and CCL22 concentrations were determined by Duoset ELISA (R&D systems, Abingdon, United Kingdom). CXCL9 and CXCL10 were detected using cytometric bead array chemokine analysis (BD Biosciences). Total protein concentration was measured using the BCA protein assay (Thermo Fisher Scientific, Göteborg, Sweden) following desalting on Zeba™ micro desalt spin columns (Thermo Fisher Scientific) and chemokine concentrations were adjusted to total protein concentrations.

### Statistical analysis

All statistical analyses were carried out in SPSS 14.0 or PRISM using the Wilcoxon signed rank test. P values less than 0.05 were considered significant.
